# Regulatory T Cells: Serious Contenders in the Promise for Immunological Tolerance in Transplantation

**DOI:** 10.3389/fimmu.2015.00438

**Published:** 2015-08-31

**Authors:** Niloufar Safinia, Cristiano Scotta, Trishan Vaikunthanathan, Robert I. Lechler, Giovanna Lombardi

**Affiliations:** ^1^MRC Centre for Transplantation, Division of Transplantation Immunology and Mucosal Biology, Faculty of Life Sciences and Medicine, King’s College London, London, UK

**Keywords:** tregs, tolerance, transplantation, cellular therapy, clinical trials

## Abstract

Regulatory T cells (Tregs) play an important role in immunoregulation and have been shown in animal models to promote transplantation tolerance and curb autoimmunity following their adoptive transfer. The safety and potential therapeutic efficacy of these cells has already been reported in Phase I trials of bone-marrow transplantation and type I diabetes, the success of which has motivated the broadened application of these cells in solid-organ transplantation. Despite major advances in the clinical translation of these cells, there are still key questions to be addressed to ensure that Tregs attest their reputation as ideal candidates for tolerance induction. In this review, we will discuss the unique traits of Tregs that have attracted such fame in the arena of tolerance induction. We will outline the protocols used for their *ex vivo* expansion and discuss the future directions of Treg cell therapy. In this regard, we will review the concept of Treg heterogeneity, the desire to isolate and expand a functionally superior Treg population and report on the effect of differing culture conditions. The relevance of Treg migratory capacity will also be discussed together with methods of *in vivo* visualization of the infused cells. Moreover, we will highlight key advances in the identification and expansion of antigen-specific Tregs and discuss their significance for cell therapy application. We will also summarize the clinical parameters that are of importance, alongside cell manufacture, from the choice of immunosuppression regimens to the number of injections in order to direct the success of future efficacy trials of Treg cell therapy. Years of research in the field of tolerance have seen an accumulation of knowledge and expertise in the field of Treg biology. This perpetual progression has been the driving force behind the many successes to date and has put us now within touching distance of our ultimate success, immunological tolerance.

## Introduction

Improvements in surgical techniques and the institution of T-cell directed immunosuppressive agents in the clinical transplantation of solid organs have seen remarkable advances now forming part of a well-established treatment for end-stage failure of several major organs. However, despite vast improvements in short-term survival rates, long-term graft survival remains poor owing to episodes of chronic rejection and the relative toxicity associated with life-long immunosuppression (1). The constant proportion of transplanted organs lost each year, necessitating re-transplantation, in a climate of donor organ shortage, places further strain on an already saturated transplant waiting list. With this in mind, the current standing of immunosuppression in transplantation is far from ideal. As such, the ultimate goal following transplantation is to induce immunological tolerance by “re-educating” the host’s immune response, permitting allograft acceptance without the need for pharmacological immunosuppression, thus ensuring long-term graft survival and abolishing drug-toxicity simultaneously.

The proposal of a distinct subset of T cells able to suppress immune responses was first put forward in the 1970s, which led to scientists around the world scouring for the existence of these “suppressor” T cells (2). It was not till the mid 90s when a thymic-derived lymphocytic population, coined regulatory T cells (Tregs), were defined. Subsequent years saw accumulating evidence certifying the therapeutic potential of these cells in preventing alloimmunity, explored in animal models, presenting Tregs as ideal candidates for use in tolerance-promoting protocols. Such an evolution in the field has since provided the impetus for the development of robust Treg manufacturing plans for the isolation and expansion of a functional and stable Treg product.

Here, we dissect the characterization and operation of these cells and outline strategies employed for their isolation and *ex vivo* expansion, which in turn have inspired their therapeutic application in bone-marrow transplantation (BMT), type-1 diabetes and, more recently, solid-organ transplantation.

## Regulatory T Cells

Tregs constitute approximately 1–3% of circulating CD4^+^ T cells in the periphery (3) and have been characterized by the high and stable expression of surface interleukin-2 receptor α chain (IL-2Rα, CD25^hi^) (4).

**Figure 1:**
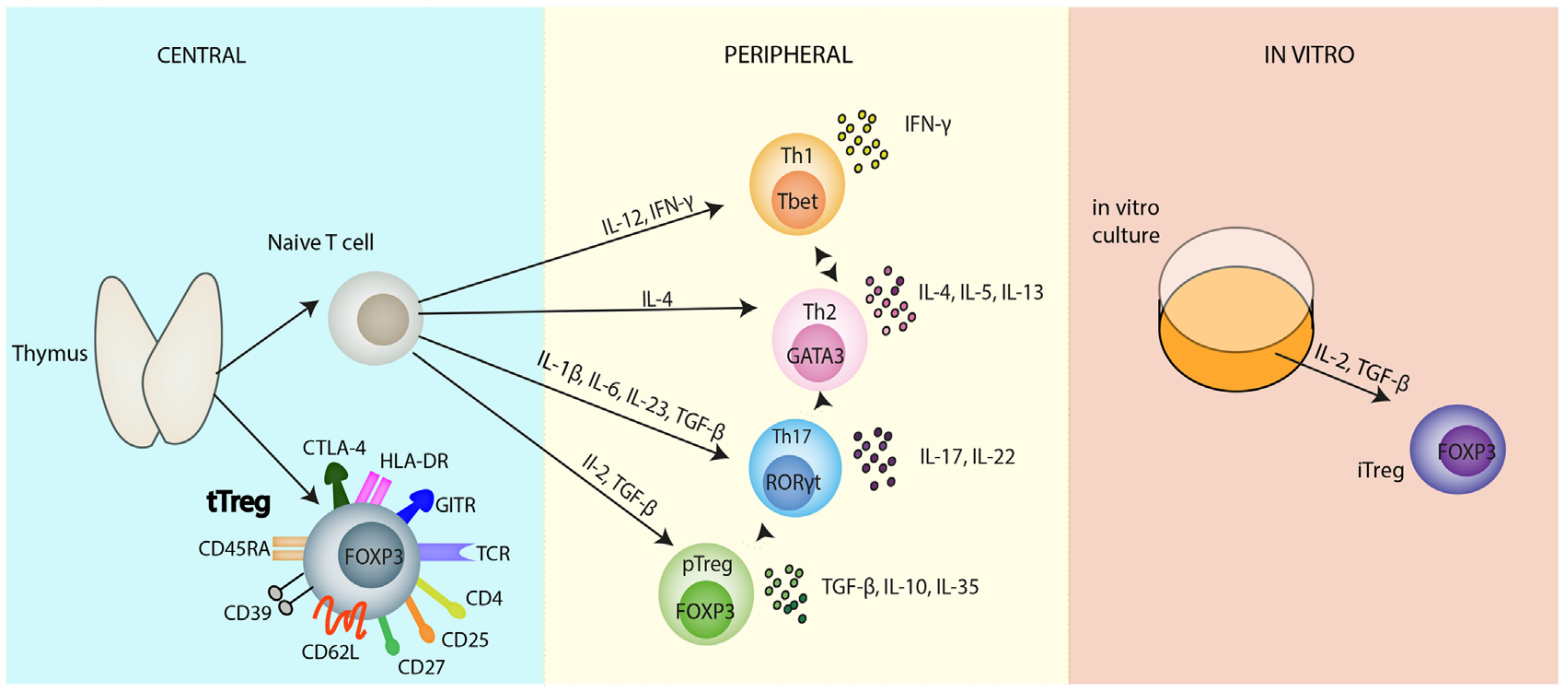
**Regulatory T cell populations**. Selection of naïve CD4^+^ T cells and natural Tregs occurs in the thymus. Thymic-derived natural Tregs (tTregs), the main focus of this review, have been reported to express a variety of activation and functional markers as depicted in the diagram. Naïve CD4^+^ T cells, subsequently, can differentiate into several different T cell subsets: Th1, Th2, Th17, induced Tregs, in the periphery, all heralding distinct immunological roles. These differentiation programs are controlled by different cytokines and each separate CD4^+^ T cell subset can be identified from their lineage-specific transcription factors responsible for the regulation and maintenance of their individual functions; T-bet (Th1 cells), GATA3 (Th2 cells), RORγt (Th17 cells), FOXP3 (Tregs). Each subset has its own immunological role *in vivo*: Th1 cells secrete IFNγ, controlling immunity to foreign pathogens. Th2 cells produce various cytokines including: IL-4, IL-5, IL-13, IL-10, which are primarily involved in promoting humoral immunity, protecting against infection. Th17 cells produce predominantly the inflammatory cytokine, IL-17, and play an important role in controlling pathogens especially at environmental surfaces and the cytokine, IL-22. Despite the apparent terminal differentiation of all these cells, they cannot be considered to be committed to one cell fate. Lineage plasticity following differentiation is depicted by the dotted arrows between the cells. This diagram is far from comprehensive; it is most likely that the future will see various changes and additions to this diagram concerning the differentiation of CD4^+^ T cells. *In vitro* generation of Tregs in the presence of IL-2 and TGF-β polarizing conditions leads to the development of iTregs. Abbreviations: APC, antigen presenting cells; CD, cluster of differentiation; CTLA-4, cytotoxic T-lymphocyte-associated protein 4; FOXP3, forkhead Box P3; IFN, interferon; IL, interleukin; IRF, interferon regulatory factor; iTreg, induced Treg; nTreg, natural Treg; pTreg, peripheral Treg; RORγt, retinoid related orphan receptor γ; T-bet, T box transcription factor; TCR, T cell receptor; TGF-β, transforming growth factor-β; Th, T helper cell; Treg, regulatory T cell.

Initially, Tregs were conventionally characterized in accordance with their site of differentiation, namely thymus-derived natural Tregs (tTregs) and peripherally induced Tregs (pTregs), alongside their *in vitro* counterparts, commonly referred to as iTregs (5) (Figure 1). tTregs, from here on referred to as Tregs, are spawned from negatively selected thymocytes, whereas the conditions favoring the generation of pTregs include suboptimal dendritic cell (DC) activation, sub-immunogenic doses of agonist peptide, mucosal administration of peptide, and antigenic encounter in a pro-tolerogenic environment, such as in the presence of interleukin-10 (IL-10), transforming growth factor-β (TGF-β), interleukin-2 (IL-2), and retinoic acid (6).

There are at least two well-defined populations of pTregs; Th3, first identified from their role in oral tolerance through the secretion of TGF-β (7), and Tr1, characterized on the basis of their role in preventing autoimmune colitis (8) and their ability to secrete large amounts of IL-10 (9, 10). As such, pTregs are implicated in the induction of oral and gut tolerance (11) and generated in chronically inflamed and transplanted tissues (12).

Of note, the phenotypic distinction of thymic and peripherally derived Tregs has not been clearly established, posing challenges in classifying the definitive proportions of these two subsets in secondary lymphoid organs and non-lymphoid tissues alike. In mice, neuropilin (Nrp-1) expressed on tTregs can differentiate these cells from their peripherally derived counterparts, which do not express this molecule (13, 14). However, this distinction does not hold true for human Tregs.

While Tregs have been crudely accrued into these populations, even within these factions, Tregs still exist in a highly organized, heterogeneous state. Various different surface and intracellular immunological markers have been studied, defining Tregs based on their functional characteristics, migration, and lineage plasticity.

In line with this, further characterization and understanding of Treg cell biology came from the discovery of FOXP3, an intracellular transcription factor known to play a crucial role in the development and function of Tregs in a highly specific manner (15). Rare mutations of the *FOXP3* gene have been linked with the development of immune dysregulation, polyendocrinopathy, enteropathy, X-linked syndrome (IPEX), leading to organ-specific autoimmune diseases including insulin-dependent diabetes mellitus and various hematological disorders (15). Furthermore, the importance of FOXP3 in the safeguarding of Treg phenotype and function has been reiterated in studies where a loss/diminution of FOXP3 expression in Tregs has been shown to affect the competency of these cells acquiring certain effector T cell properties, including production of cytokines, such as IL-2, IL-4, IL-17, and IFN-γ (16).

Additionally, while *FOXP3* has been termed a “master control gene,” specifically with regards to Treg development, its expression is not uniformly homogenous. In contrast to mice, where *foxp3* is expressed exclusively on Tregs, in humans, increasing evidence has shown that effector cells can transiently express FOXP3, with no associated regulatory activity. Based on such studies and taking also into account its intracellular expression, this marker in isolation cannot be considered to be entirely sufficient in demarcating human Tregs (17).

However, reports have commented on the inverse correlation between the expression of the α-chain of the IL-7 receptor, CD127, and FOXP3 expression with respect to Treg functional suppressive capabilities (18). As such, the combination of CD25, FOXP3, and CD127 are considered to be the most stringent markers in defining Tregs in the research setting.

**Figure 2:**
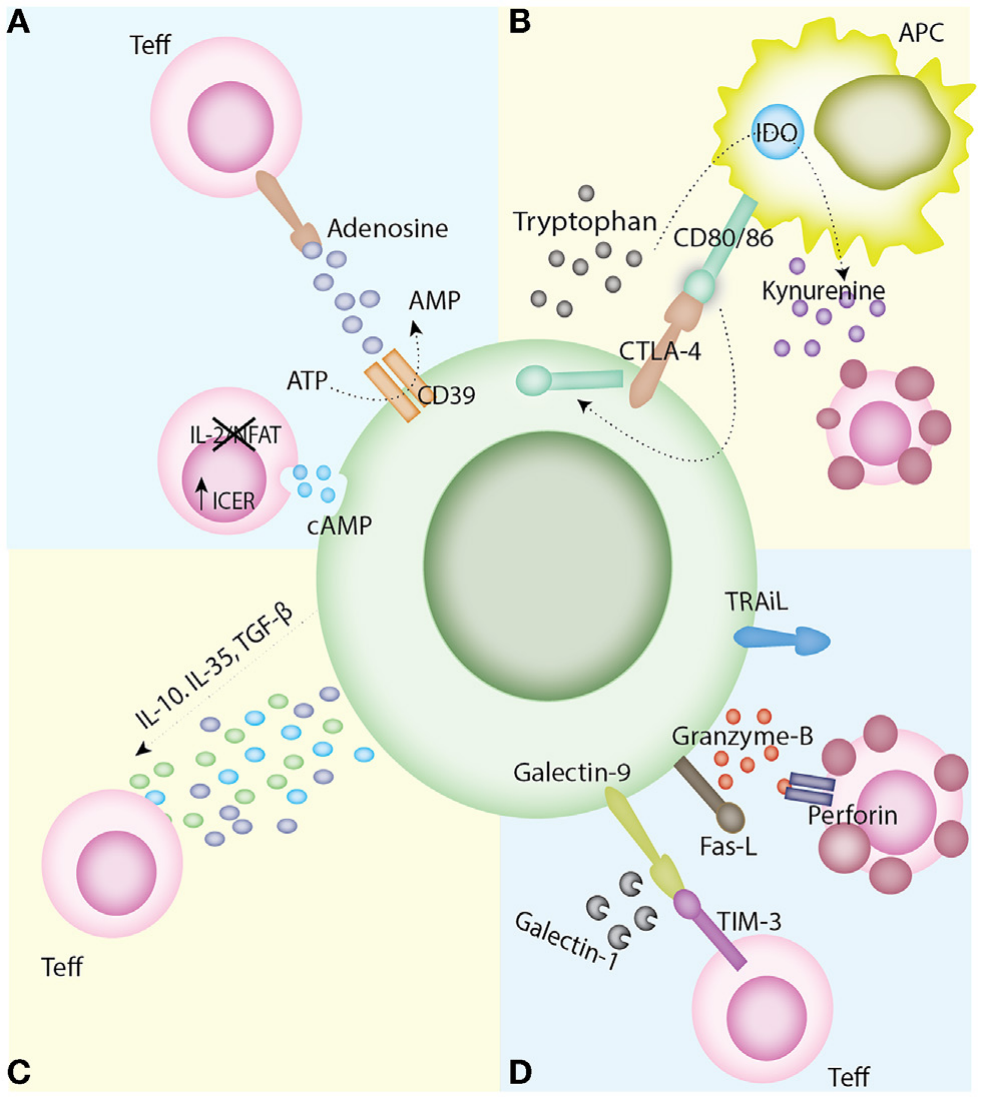
**Mechanisms of Treg suppression**. **(A)** Disruption of metabolic pathways. The ectoenzymes CD39 and CD73, expressed on Tregs, result in the metabolism of ATP to AMP and in turn producing the immunoregulatory purine, adenosine. Tregs have also been found to express high levels of intracellular cAMP. This is transferred to T effector cells through gap junctions, which leads to the upregulation of ICER and in turn the inhibition of NFAT and Il-2 transcription leading to apoptosis by IL-2 deprivation. **(B)** Modulation of APC maturation and function. The interaction of CTLA-4 on Tregs with its ligand CD80/86 on APCs delivers a negative signal for T cell activation. CTLA-4’s mechanism of action is varied including the capture of its APC-expressed ligands and subsequent trans-endocytosis and also the upregulation of IDO and the generation of kynurenines. **(C)** Anti-inflammatory cytokine production. The secretion of anti-inflammatory cytokines, such as IL-10, IL-35, and TGF-β, has been linked with inhibition of T cell activation *in vivo*. **(D)** Induction of apoptosis. Tregs have the capacity to directly induce apoptosis via granzyme A/B and perforin, TRAIL, the Fas/Fas-ligand pathway, the galectin-9/TIM-3 pathway, or the production of galectin-1. Abbreviations: APC, antigen presenting cell; AMP, adenosine monophosphate; ATP, adenosine triphosphate; cAMP, cyclic adenosine monophosphate; CD, cluster differentiation; CTLA-4-cytotoxic T lymphocyte antigen-4; DC, dendritic cells; ICER, inducible cAMP early repressor, IDO, indoleamine 2,3-dioxygenase IL, interleukin; NFAT, nuclear factor of activated T cells; TGF-β, transforming growth factor-β; TIM-3, T cell immunoglobulin and mucin domain-3; TRAIL, tumor necrosis factor-related apoptosis-inducing ligand; Treg, regulatory T cells.

Additionally, following the recent discovery of naïve suppressive FOXP3^+^ cells (CD45RA^+^) present in the cord blood and in adult blood, and FOXP3^+^ cells, which express a memory-like phenotype (CD45RA^−^), it has been proposed that three phenotypically and functionally distinct sub-populations based on the differential expression of CD25, FOXP3, and CD45RA can be defined: population I (CD25^++^FOXP3^+^CD45RA^+^) classified as resting Tregs, population II (CD25^+++^FOXP3^hi^CD45RA^−^) termed activated Tregs, and population III (CD25^++^FOXP3^+^CD45RA^−^), which was proposed to consist of non-suppressive FOXP3^lo^ cells (19). Further analysis of the three populations by Miyara et al. revealed that population I and II were both able to suppress *in vitro* with population II displaying a higher expression of cytotoxic T-lymphocyte-associated protein 4 (CTLA-4), a mechanism proposed for Treg suppressor function (Figure 2), yet were more prone to apoptosis following exertion of their suppressive function. Population III, however, was shown to be non-suppressive *in vitro* (19).

Demarcation of these three populations of Tregs was also able to depict the differentiation dynamics of FOXP3^+^ Tregs *in vivo*. Resting Tregs were found to upregulate their FOXP3 expression, following stimulation, and mature to terminally differentiated activated Tregs thus replenishing the apoptotic pool of activated Tregs. Miyara et al. suggested that population III had the greatest potential to differentiate into inflammatory Th17 cells, inferred from their relative IL-17 production following cytokine stimulation. The three comparative populations are found in different proportions in certain biological environments and their analysis can prove to be instrumental in identifying the immunological pathophysiology of disease and the optimal Treg subpopulation for cell therapeutic application.

It should, however, also be noted that the definitive functional characteristics of population III are controversial. Booth et al. and data from our laboratory indicated that both CD45RO^+^ and CD45RA^+^ Treg subsets are equally suppressive, population III representing a *bona fide* Treg subpopulation, bearing T cell memory markers (20). Moreover, we and others, have also reported the expression of CD161, a member of the killer cell lectin-like receptor subfamily B, on a subpopulation of human Tregs in population III, that produce IL-17 upon *in vitro* activation in the presence of IL-1β, but not IL-6. In addition, evidence has also supported the suppressive capacity of these cells (21, 22).

Above, we have outlined some of the key Treg markers of which are pertinent when considering the isolation of these cells for clinical application. However, one must be wary that the array of markers outlined in this review is far from exhaustive. For a comprehensive review of Treg markers, the reader is directed to Schmetterer et al. (23) and Povoleri et al. (6).

The mechanisms of Treg suppression still remain elusive. *In vitro* studies have demonstrated that the immunosuppressive qualities native to Tregs manifest through a variety of mechanisms, namely, modulation of APC maturation and function (24–26), anti-inflammatory cytokine production (27–30), induction of apoptosis in target cells (31, 32), and disruption of metabolic pathways (33, 34) (Figure 2).

## Regulatory T Cells in Transplantation; Lessons Learnt from Pre-Clinical Data

The current paradigm hypothesizes that immune tolerance in transplantation is determined by a balance of Tregs over T effector cells. With this phenomenon in mind, the therapeutic potential of inducing and expanding Tregs directly *in vivo* or infusing autologous *ex vivo*-expanded Tregs represents a promising approach in the induction and maintenance of transplantation tolerance.

Series of pre-clinical rodent models of skin and cardiac transplantation demonstrated that Tregs present in the recipient at the time of transplantation are critical in the induction and maintenance of tolerance [reviewed in Ref. (35)]. Additionally, mouse models of BMT further supported the importance of adoptive Treg therapy, whereby the transfer of freshly isolated Tregs together with the bone-marrow allograft resulted in amelioration of graft versus host disease (GvHD) and facilitated engraftment (36, 37).

Moreover, adoptive transfer of Tregs has been shown to prevent rejection in other murine models of transplantation, such as pancreatic islets (38).

An issue for consideration in Treg cell therapy in transplantation is the relevance of Treg allospecificity with the selective advantage that the immunomodulatory function of these cells would be concentrated at the site of alloantigen and immune activation (39). An additional advantage of alloantigen-specific cellular therapy is that undesirable pan-suppression, resulting in increased risk of infections and cancers, is less likely to occur.

Although the indirect pathway has been implicated in acute graft rejection (40), its influence has been more closely associated with chronic allograft rejection (41). Indeed, much evidence suggests that for tolerance to occur, this is the pathway that needs to be regulated and it is this pathway of allorecognition that is used by Tregs for immunoregulation (29, 41–44).

In agreement, we have shown that using Tregs with indirect pathway anti-donor allospecificity for a single MHC class I result in the induction of donor-specific transplantation tolerance in a murine skin transplant model following thymectomy and selective T cell depletion (45). However, in a later study, we reported that Treg lines specific for directly and indirectly presented alloantigens are needed to induce indefinite survival of MHC-mismatched heart allografts, with Tregs with indirect allospecificity necessary to prevent chronic vasculopathy (46). Moreover, Joffre et al. have provided additional evidence that Tregs with direct allospecificity alone cannot protect against chronic rejections (47) supporting the notion that Tregs with both specificities are necessary to control allograft rejection.

Additional support for the use of alloantigen-specific Tregs in the transplant setting has been made available by the use of currently available humanized mouse models of allotransplantation (48–52). These models are based on the reconstitution of immunodeficient mice with human immune cells. Our group has recently shown the efficacy of human Tregs with direct allospecificity in preventing alloimmune dermal tissue injury using a humanized mouse model of skin transplantation in which only T cells have engrafted (53). The general consensus throughout these studies concluded that donor antigen-specific Tregs are more effective as compared to polyclonal Tregs.

In addition to the evidence supporting the importance of antigen-specific Tregs in preventing solid-organ rejection, after BMT donor-specific Tregs have been shown to preserve graft versus tumor activity, while inhibiting GvHD (54). However, further studies in the context of GvHD have reported that the transfer of Tregs enriched for alloantigen-specificity showed only moderately improved efficacy when compared to polyclonal Treg cell populations (55). As such, phase I clinical trials, using polyclonal Tregs, following hematopoietic stem cell transplantation have been conducted (56–58).

Such adoptive transfer experiments in rodents, therefore, have informed and instigated efforts to harness the immunoregulatory properties of these cells in novel tolerance promoting strategies in the prevention of rejection after organ transplantation. Thus, “tipping the balance” in favor of regulation by directly applying *ex vivo*-expanded Tregs is a promising strategy. In the next section, we will highlight the advances in the field in view of Treg isolation and expansion, reviewing some of the challenges and progress to date as well as review the lessons learned from the clinical application of these cells.

## Treg Manufacture for Clinical Application

### Approaches for Treg isolation

The effective implementation of Treg therapy in transplantation is dependent on Treg manufacturing plans with protocols that are compliant with good manufacturing practice (GMP).

The clinical Treg selection protocol to date in the UK has been used by our group in the new Clinical Research Facility (CRF) at Guy’s Hospital and involves a combination of depletion of CD8^+^ cells and positive selection of CD25^+^ cells using the automated CliniMACS plus system (Miltenyi Biotec, Bisley, United Kingdom), which is centered around the concept of magnetic bead isolation. The major drawback with such a technique is that this process does not allow the selection of Tregs based on multiple parameters, an attractive prospect when trying to isolate a Treg population with the desired characteristics for cell therapeutic application.

Additionally, the lack of distinction of CD25^hi^ cells, using this protocol, means that the isolation may include contaminating conventional activated T cells, explaining the reduced purity of this strategy for Treg isolation as compared to the use of the fluorescence-activated cell sorting (FACS) technique (59, 60).

Despite the CliniMACS system being the only currently available GMP compatible technology for the isolation of clinical grade Tregs in the UK, there is still much enthusiasm for clinical cell separation using FACS sorting. Although currently unclear as to which Treg subset provides the best therapeutic activity, GMP-compliant FACS sorting will open up the possibility of isolating Treg subsets with potent suppressive function, specificity, and those that are epigenetically stable.

Two different combinations of markers have been proposed to be promising for the isolation of a pure Treg population. The first seeks to isolate CD4^+^CD25^hi^ Tregs with the addition of an antibody to select for CD45RA^+^ cells and so eliminate antigen experienced or memory T cells (61). Moreover, this so-called naïve Treg population yields Tregs with a greater suppressive capacity than total CD25^hi^ cells (62) and have the greatest expansion potential (61).

Furthermore, it has been demonstrated that between the *FOXP3* promoter and the first exon lies a stretch of highly conserved, non-coding sequence that is differentially methylated in tTregs, pTregs, and T effectors (63, 64). This sequence, referred to as the Treg-specific demethylated (TSDR) region, is crucial at maintaining high FOXP3 expression in Tregs (65).

Additionally and in support of the isolation of the CD45RA^+^ Treg subset for cell therapy application, supplementary evidence report that after 3 weeks of *in vitro* expansion, the CD45RA^+^ expanded Tregs remained demethylated at the TSDR region, confirming their stability during expansion (60, 62).

The importance of isolating and expanding a stable Treg population becomes even more pertinent when considering Treg cell therapy in inflammatory/autoimmune conditions. Emerging data have highlighted that despite the strict government of FOXP3 expression, Tregs can downregulate FOXP3 in the presence of inflammatory cytokines. In agreement, Yang et al. have shown that exposure of Tregs to IL-6 and IL-1 *in vitro* results in the expression of IL-17 (66). *In vivo*, loss of FOXP3 has also been documented in the setting of autoimmune disease (67) fetal acute infections (68), TLR stimulation (69), and homeostatic proliferation (70).

Therefore, with evidence supporting the stability of CD4^+^CD25^+^CD45RA^+^ Tregs, we have recently advocated the isolation and expansion of these cells for cell therapeutic application in the setting of inflammatory bowel disease (71).

Despite such studies in favor of CD45RA^+^ cells, one drawback is that the number of naïve Tregs decline in the peripheral blood with age (72) and hence isolation based on this approach may prove to be impractical.

The second approach still uses the fundamental CD4^+^CD25^hi^ phenotype to isolate Tregs but also includes CD127 expression. The rationale placed on the foundation that in human Tregs, there is a reciprocal expression of CD127 and FOXP3 and thus CD127 provides a sortable surrogate marker for FOXP3^+^ Tregs (18). Moreover, two elegant studies (48, 50) support the *in vivo* superiority of the CD4^+^CD25^+^CD127^lo^ Tregs in regulating alloreactivity compared to Tregs isolated based on the expression of CD4 and CD25 alone.

Such studies merely highlight the importance of multiparameter separation of Tregs, using the FACS cell sorter. In this regard, the last few years have not only seen significant efforts made in obtaining the relevant regulatory approvals to integrate the FACS cell sorter into a clinical cell production process, but an immense progress in the technology to do so.

As such, a new era of clinical flow sorting has seen the recent introduction of a class of flow sorting devices, utilizing microfluidic chips instead of the classical flow-in-air droplet sorters. Unlike traditional sorting, there is no high-pressure, shearing forces, or dilution by sheath fluid, resulting in a cell processing that may preserve cell function and viability. Additionally, cells are processed in closed systems, thus eliminating the risk of contamination of the product during processing. Such a system, however, did not present itself without technical challenges including the initial slow sort speed. These have been overcome either by massive parallel sorting on a single microfluidic chip, such as in the Cytonome GigaSort System (Cytonome/ST, LLC, Boston, MA, USA) (73) or by the introduction of mechanical microvalves operating at high speed, such as the recently introduced MACS Quant Tyto (Miltenyi Biotec, Bergisch Gladbach, Germany).

Although not yet clinically approved, both machines are in principal designed for clinical cell separation processes and provide significant advantages allowing their integration into GMP-compliant production processes.

### Polyclonal Treg expansion: Optimization of current culture conditions

One of the obstacles in the implementation of clinical protocols for adoptive Treg cell therapy is their relative paucity in the circulation. This means that for cellular therapy, it will almost certainly be necessary to expand these cells *ex vivo*, to clinically relevant numbers, prior to their administration. It has already been demonstrated that Tregs can be readily expanded using anti-CD3/CD28-coated beads, supplemented with IL-2 (60, 74). However, under these circumstances, effector cells have the potential to proliferate vigorously, posing a major problem for MACS-purified CD4^+^CD25^+^ Tregs, as they are often contaminated with CD25^+^FOXP3^-^ cells. Thus, this not only puts under question the potential safety of the final product but also the efficacy. As such, much effort has, therefore, focused on optimization of culture conditions to ensure the expansion of Tregs to achieve the necessary numbers yet limit the potential expansion of contaminant cells.

#### Rapamycin

**Figure 3:**
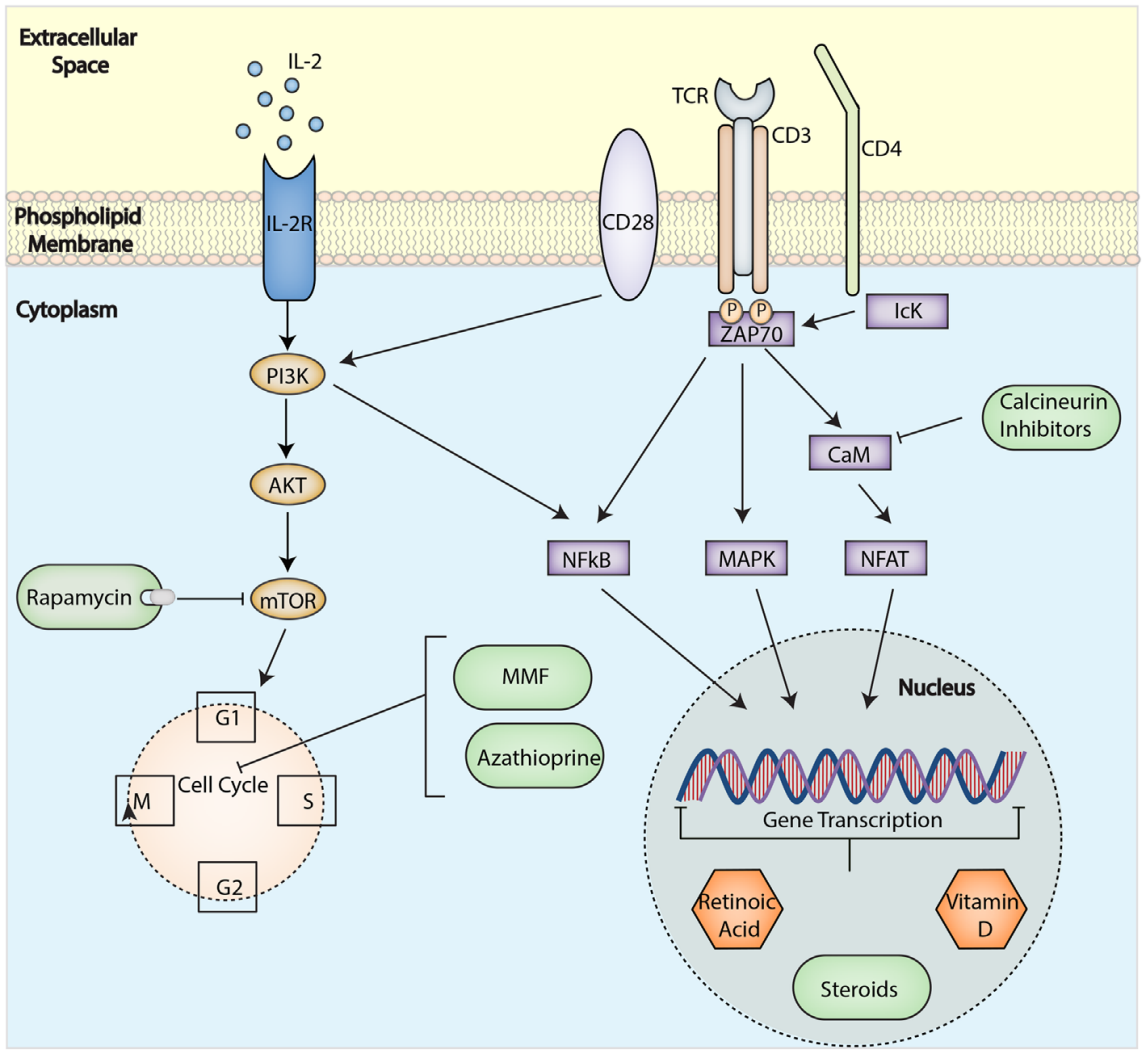
**Immunological targets for immunosuppressive drugs in T cells**. Drugs can affect specific molecular pathways and control gene expression to block alloactivation of T cell after transplantation and induce tolerance. Abbreviations: TCR, T-cell receptor; mTOR, mammalian target of rapamycin; MMF, mycophenolate mofetil; PI3K, phosphatidylinositol 3-kinase; IL-2R, interleukin-2 receptor; NFkB, nuclear factor κ-light-chain-enhancer of activated B cells; MAPK, mitogen-activated protein kinase; NFAT, nuclear factor of activated T-cells.

This immunosuppressant mechanism of action involves the inhibition of the mammalian target of rapamycin (mTOR), which is downstream of phosphatidylinositol 3-kinase (PI3K), a signaling molecule activated by CD28 or IL-2 receptor engagement in T cells (75) (Figure 3). Characteristically, IL-2 receptor engagement activates both PI3K-mTOR and Janus kinase-STAT pathways. However, biochemical analysis of IL-2 signaling in Tregs has shown that the PI3K–mTOR pathway is underactive, whereas the Janus Kinase–STAT pathway remains intact, suggesting that Tregs preferentially signal through the latter in turn conferring their resistance to mTOR inhibition (76). In agreement, genetic ablation and cellular experiments that demonstrate mTOR deficiency or the addition of rapamycin favor the growth and preserved function of Tregs (77, 78). Paralleling these *in vitro* observations, it has been shown that rapamycin can potentiate the ability of Tregs to inhibit transplant arteriosclerosis in a humanized mouse system (79). Furthermore, in transplant patients, the use of rapamycin-based immunosuppression is also associated with an increased proportion of Tregs as compared to patients on calcineurin inhibitors (CNI) (80, 81). Thus, by favoring Treg survival and expansion and by preventing the outgrowth of contaminating effector T cells (76, 82), rapamycin ensures the selection of a pure Treg population.

Further research has not only confirmed the unique preferential preservation of Tregs by rapamycin but has also reported its role in ensuring Treg stability. Treatment of CD4^+^CD25^hi^FOXP3^+^ with rapamycin has been shown to inhibit the development of IL-17-producing cells and to maintain a stable Treg phenotype favoring the expansion of non-plastic Treg subsets, both *in vitro* and *in vivo* (83–85). The mechanism by which this occurs is thought to involve alterations in the epigenetic profile allowing for the active transcription of *FOXP3* (84, 86, 87). These findings support the use of rapamycin in clinically applicable protocols for the expansion of human Tregs. We are currently testing the safety of Tregs expanded following this protocol in the CRF at Guy’s Hospital in two Phase I/II clinical trials: in kidney (ONE Study: NCT02129881) and liver (ThRIL: NCT02166177) transplant patients.

#### Retinoic Acid and Vitamin D

Despite recent advances in Treg biology, large-scale manufacture of these cells remains challenging in view of studies reporting that even highly pure Tregs lose FOXP3 expression over time. In line with this, a study by Hoffman et al. further concluded a loss of FOXP3 expression by Tregs upon repeated anti-CD3/CD28 stimulation in culture, in turn forfeiting their stability (62). As such, recent attention has been drawn to other approaches that ensure Treg stability in culture, an example including the supplementation of cultures with all-*trans-*retinoic acid (ATRA).

Of note, natural derivatives and metabolized products of vitamin A, such as β-carotene, retinol, retinal, isotetrinoin, ATRA, and 9-*cis*-Retinoic acid all have important roles in cell differentiation, growth, and apoptosis (88). ATRA is one of the most influential molecules on T cells and has been reported to affect T cell fate by contributing to Th1/Th17 as well as Treg differentiation (89). In combination with TGF-β, ATRA has been shown to promote differentiation of naive murine and human T cells into Tregs (90–92) and, more recently, as a treatment to expand human Tregs and increase their function (84, 85, 93, 94). The molecular pathway by which ATRA favors the expansion of Tregs is not entirely clear, but it is thought to induce chromatin de-condensation recruiting histone acetyl-transferases and transcription machinery to the *FOXP3* promoter (95, 96). Furthermore, experiments in animal models have shown that deletion of ATRA nuclear receptor results in significant loss of FOXP3 expression in Tregs, suggesting that ATRA may act to stabilize FOXP3 expression (97).

However, while the use of ATRA during Treg culture heralds the expansion of a highly suppressive Treg population for cell therapy (84, 85, 94), there are concurrently major concerns regarding its use, primarily since ATRA preferentially stimulates *ex novo* generation of iTregs, which as eluded to earlier, have an unstable genetic phenotype with their plasticity influenced *in vivo* by the inflammatory conditions (87). As a consequence, the regulatory phenotype induced *in vitro* may be easily reverted into a pro-inflammatory one upon *in vivo* administration (98).

Indeed, there is a dynamic relationship between the transcription factors RORγT and FOXP3 in T cells that rules the balance between Th17 cells and Tregs. The competitive antagonism of these trans-acting factors is controlled by pathways downstream of either IL-2 (pro-Treg) or IL-1β/IL-6 signaling (pro-Th17) (98). For this reason, an intense inflammatory condition may fully override ATRA-mediated FOXP3 expression and revert a protective Treg preparation into a potentially harmful immune response (87, 98).

Another potential supplementary candidate in Treg expansion that has attracted much attention is Vitamin D, with growing evidence supporting the immunomodulatory roles of this vitamin and its importance in the induction and maintenance of FOXP3^+^ Tregs *in vivo*. As such, studies have highlighted that serum concentrations of vitamin D positively correlate with the number and frequency of FOXP3^+^ Treg cells in the peripheral blood of patients (99–102). However, the mechanism by which vitamin D controls the generation/expansion of Tregs is not completely clear, with vitamin D concentrations playing differing roles (102, 103). It appears that high non-physiological concentration (10^−6^ M) of Vitamin D induces IL-10 production by CD4^+^ T cells, providing an explanation for the immune-modulating and Treg-promoting ability of these settings. Instead, at a more physiological concentration (10^−7^ M), vitamin D, together with TGF-β, favors the expansion of a highly suppressive FOXP3^+^ Tregs (102, 103).

The past few years have seen considerable effort focused on defining an optimal technique to isolate and expand Tregs from peripheral or cord blood. The studies outlined above highlight the future research directions in view of devising protocols that incorporate the use of these reagents in the expansion of an optimal Treg population for cell therapy application.

## Alloantigen-Specific Regulatory T Cell Expansion

In view of the wealth of animal data from our laboratory (49, 53, 104) and others (47, 48, 55) in support of the importance of antigen-specific Tregs in the setting of solid-organ transplantation, efforts have been directed at the generation of antigen-specific Tregs for cellular therapy in this setting.

To date, the generation and expansion of alloantigen-specific Tregs have proved to be an arduous task, in particular Tregs with indirect allospecificity. Recently, there have been advances in the propagation of Tregs with direct allospecificity using donor APCs, such as DCs and unfractionated peripheral blood mononuclear cells (PBMCs) (53, 105–107). In this regard and in a previous study, we screened human Tregs for activation markers following stimulation with allogeneic peripheral blood or dermal CD1c^+^ DCs (53). We subsequently defined the upregulation of CD69 and CD71 by Tregs, at 3–5 days after activation, delineating the alloantigen-specific Tregs. Furthermore, we were able to comment on the suppressive superiority of these antigen-specific Tregs as compared to polyclonally expanded Tregs.

Moreover, recent reports have highlighted the effectiveness of CD40L-activated B cells in the induction and expansion of antigen-specific Tregs *in vitro* (108–110). As such and in collaboration with colleagues at University of California (UCSF), we have shown the clinical grade manufacture of Tregs against allogeneic human leukocyte antigen (HLA) of the donor (104). Purified Tregs were stimulated *ex vivo* with CD40L-activated allogeneic B cells, followed by subsequent expansion using anti-CD3/anti-CD28-coated beads with the addition of IL-2. By employing this protocol, we were able to demonstrate not only the successful expansion (300- to 500-fold) but also the alloreactivity of the expanded Tregs against the donor antigen. We further demonstrated the antigen-specific suppressive function of the expanded Tregs in protecting against alloimmune-mediated skin damage in a humanized mouse model of transplantation. The manufacture of Tregs, using this protocol, is currently being tested as part of a clinical trial at UCSF (NCT02188719).

An alternative approach has been to engineer Tregs with the ability to target specific antigens by expressing antigen-specific TCRs (111) or taking advantage of the growing field of Chimeric Antigen Receptors (CAR) (112). To date, most of the CAR studies have focused on tumor antigen-specific cytotoxic CD8^+^ T lymphocytes for the treatment of human cancers. However, several studies in animals suggest that CAR-expressing Tregs can be efficacious in preventing experimental autoimmune encephalitis (EAE) (113) or colitis (114). More recent work from our group has set out to investigate the efficacy of CAR-expressing Tregs in the setting of transplantation.

It is also pertinent to note that given the experimental evidence detailing the synergy of direct and indirect Tregs in the setting of transplantation tolerance, considerable efforts have been concentrated on generating and expanding Tregs with indirect allospecificity to further assist in this endeavor (115, 116). In contrast to the definitive selective expansion of Tregs with direct allospecificity from the existing repertoire, studies have demonstrated the difficulties in generating Tregs with indirect allospecificity using the same APCs used for the generation of Tregs with direct allospecificity. TCR-transduction was used by us to confer indirect allospecificity and we have demonstrated both in skin and heart transplants, the efficacy of Tregs with such a specificity. In addition, we have shown the advantage of conferring indirect alloreactivity during the expansion of direct alloreactive Tregs so as to generate Tregs with dual specificity, with promising results (111, 117).

Despite the on-going efforts to develop protocols for the manufacture and *ex vivo* expansion of allospecific Tregs, it is pertinent to note that our initial data highlighted that a highly pure population of Tregs was essential prior to their allospecific *ex vivo* expansion for this to be a success (104). As such and in view of the lack of GMP-compliant sorting technology in the UK, the application of antigen-specific Tregs in trials of Treg cell therapy in the UK have not been possible. However, with the upcoming installation of a GMP-compliant cell sorter into our facilities, efforts will be directed toward the generation of an optimal precursor population of antigen-specific Tregs for cellular therapy in the near future.

## Tregs Therapy in Solid-Organ Transplantation; Our Experience to Date

The results of the trials to date have highlighted the favorable safety profile of freshly isolated and polyclonally expanded Tregs with varied reports of efficacy (56–58, 118) (Table 1). As a result, the prospects of Treg adoptive cell therapy are now widely recognized with the information gleaned from these preliminary trials now guiding the clinical progression of these cells into the realms of organ transplantation.

**Table 1:** **Clinical trials of Treg immunotherapy**.

Clinical trial number	Investigators	Setting	Patients recruited	Isolation	Treg doses	Study overview and results	Reference
N/A	Trzonkowski et al.	GVHD adult	2	FACS: CD4^+^CD25^+^CD127^−^	1 × 10^5^ to 3 × 10^6^/kg	The first patient had chronic GvHD 2 years post BMT. After receiving 0.1 × 10^6^/kg FACS purified *ex vivo* expanded Tregs from the donor, the patient was successfully withdrawn from immunosuppression without evidence of recurrence. The second patient had acute GvHD at 1-month post transplantation, treated with several infusions of expanded donor Tregs. Despite the initial and transitory improvement, the disease progressed and ultimately resulted in the patient’s death	(58)
NCT00602693	Brunstein, McMillan, Blazar (2010)	GvHD adult	23	CliniMACS: CD25^+^	0.1, 0.3, 1, and 3 × 10^6^/kg	Tregs were isolated from a third party UCB graft and expanded polyclonally with anti-CD3/CD28 coated beads and recombinant IL-2 over a period of 18 days. Patients received expanded Tregs at doses ranging from 1 × 10^5^/kg to 30 × 10^5^/kg. Targeted Treg dose was only achieved in 74% of cases. Compared with the 108 historical controls, there was a reduced incidence of grades II–IV acute GvHD (from 61–43%; *p* = 0.05), although the overall incidence of GvHD was not significantly different.	(56)
N/A	Di Ianni et al.	GvHD adult	28	CliniMACS: CD4^+^CD25^+^	2–4 × 10^6^/kg	Patients received donor Tregs without *ex vivo* expansion and donor effector T cells (Teff) without any other adjuvant immunosuppression. Different dose regimens were used, ranging from 5 × 10^5^/kg Teffs with 2 × 10^6^/kg Tregs to 2 × 10^6^/kg Teffs with 4 × 10^6^/kg Tregs. As two patients receiving the latter regimen developed acute GvHD, compared with none of the other patients, the dose of 1 × 10^6^/kg Teffs with 2 × 10^6^/kg Tregs was reported to be safe. Patients receiving Tregs demonstrated accelerated immune reconstitution, reduced CMV reactivation, and a lower incidence of tumor relapse and GvHD when compared to historical controls. Disappointing patient survival was reported with only 13 out of the 26 patients surviving	(57)
N/A	Marek-Trzonkowska et al.	Type-I diabetes children	12	FACS: CD4^+^CD25^+^CD127^−^	10–20 × 10^6^/kg	One year follow-up of 12 children with Type-I diabetes, treated with autologous-expanded *ex vivo* Tregs. Patients received either a single or double Treg infusion up to a total dose of 30 × 10^6^/kg. The data supported the safety of the infused Tregs, with 8/12 treated patients requiring lower requirements of insulin, with two children completely insulin independent at 1 year	(118)
NCT01210664	Bluestone et al.	Type-I diabetes adult	14	FACS: CD4^+^CD25^+^CD127^−^	5 × 10^6^–2.6 × 10^9^/kg	Infusion of 14 type-I diabetic patients with *ex vivo-*expanded Tregs (FACS purified and two rounds of anti-CD3/anti-CD28 stimulation). The first cohort of patients received 0.05 × 10^8^ cells, the second: 0.4 × 10^8^ cells, the third: 3.2 × 10^8^ cells, and the fourth: 2.6 × 10^9^ cells. Enrolment and infusion is complete	Bluestone, in preparation

*UCB, umbilical cord blood; GVHD, graft versus host disease; BMT, bone marrow transplantation*.

In this regard, Yamashita et al. recently reported the first trial of donor alloantigen-specific Tregs in patients undergoing living donor liver transplantation (119). Here, iTregs were generated whereby recipient PBMCs were co-cultured with irradiated donor PBMCs in the presence of costimulatory blockade. Subsequent administration of 0.6–2.6 × 10^9^ iTregs in splenectomized patients, concurrently receiving cyclophosphamide, was found to be not only safe in this setting but also enabled the withdrawal of immunosuppression in 6 out of the 10 patients recruited.

As alluded to earlier, the last few years has also seen the start of two clinical trials of Treg immunotherapy in solid-organ transplantation at King’s College London, the ONE Study (NCT02129881) and ThRIL (NCT02166177).

The ONE study is a multicenter Phase I/II study funded by the European Union FP7 program, investigating the safety of and potential efficacy of infusing *ex vivo*-expanded Tregs, among other regulatory cells. It is a dose escalation trial, designed to assess doses of 1, 3, 6, 10 × 10^6^ Tregs/kg, injected at 5 days after transplantation so as to determine the maximum-tolerated dose. Of importance, patients, receiving the cell products at the different centers will be on a similar immunosuppression regimen that includes prednisolone, tacrolimus, and mycophenolate mofetil (MMF). This will enable the direct comparison of transplant outcomes between the varied cell products tested as part of the ONE study.

Although the primary goal of the ONE study is to assess safety, the production feasibility of each of the cell products, at the varied doses, will also be assessed. In this regard, we have successfully produced the final product that to date has enabled three patients to be dosed at 1 × 10^6^/kg, a further three patients at 3 × 10^6^/kg and one patient at 6 × 10^6^/kg. Five more patients remain to be dosed, two at 6 × 10^6^ and three at 10 × 10^6^. The final Treg product manufactured by us has been used to treat patients recruited at Oxford University and by Guy’s Hospital.

ThRIL (NCT02166177) is a combined Phase I/IIa clinical trial of Treg immunotherapy in the setting of liver transplantation where the safety, tolerability, and efficacy of 1 × 10^6^/kg and 4.5 × 10^6^/kg of polyclonally expanded Tregs will be assessed with thymoglobulin and an mTOR-inhibitor-based immunosuppression regimen. ThRIL is currently in the recruitment stage and to date, we have dosed the first patient for this trial.

The near future will see the reporting of these trials, which will focus primarily on the safety of the injected cells, but will also speculate on their relative therapeutic efficacy, with reference to graft survival and supplementary biochemical and immunological markers of tolerance, in a bid to support larger Phase II/III studies. The success of such trials and the outlook of Treg therapy as an entirety will be defined from effective and informative clinical trial designs with adherence to hard efficacy end points. Thus, key issues will need to be addressed prior to the design of such trials including adjunct immunosuppressive regimens, the timing and number of injections, the dose of Tregs with the desired specificity, and the trafficking properties of the infused cells.

## Tregs Immunotherapy; Future Directions

### Immunosuppression and Tregs

Despite the initial confidence in adoptive Treg cell therapy as a self-sufficient entity, experimental data have shown that the efficacy of Treg therapy requires a favorable *in vivo* environment, supporting both cell engraftment and the chance of inducing tolerance, such as transient host T cell depletion instituted following immunosuppressive treatments (120, 121).

As already discussed, the immunosuppression regimen for the ONE study includes the combination of CNI, tacrolimus together with prednisolone, and MMF. The main question that arises next is how this microenvironment will influence the Tregs *in vivo* following adoptive transfer.

In this regard, studies have shown that the use of CNIs during adoptive Treg therapy may have an indirect impact on the survival and suppressive ability of Tregs in view of the strong dependence of these cells on the exogenous supply of IL-2 (122, 123).

In agreement, studies in animal models have reported that CNI treatment reduces FOXP3 expression in natural Tregs (123, 124), diminishes the frequencies of CD4^+^CD25^+^FOXP3^+^ T cells (81), and fails to support the differentiation of the highly suppressive CD4^+^CD25^+^CD27^+^ Treg subset upon alloantigen stimulation (125). Studies in humans confirmed the negative effect of this treatment on Tregs, suggesting that the continuous CNI therapy is linked with progressive decline in Treg numbers (126). However, despite these major direct drawbacks on Tregs, it may be worth considering that CNI therapy may be still used to set up a favorable environment before the Treg infusion or at sub-therapeutic doses in combination with other drugs during Treg therapy. In this regard, Wang and collaborators showed that kidney transplanted recipients treated with MMF and low-dose tacrolimus had an induction of CD4^+^CD25^+^FOXP3^+^ Tregs that could expand in the periphery and accumulate in the allograft. Additionally, the *in vitro* analysis of these cells confirmed the maintenance of their suppressive function (93).

On the other hand, the effects of MMF on Tregs have not been extensively analyzed and the few results reported are controversial. Data in literature support the idea that the influence of this drug on cell division may alter the expansion of antigen-specific Tregs and prevent the settlement of a long-term tolerance. In line with this notion, MMF administration in a murine model significantly inhibited the expansion of OVA-specific CD4^+^CD25^+^Foxp3^+^ Tregs after OVA immunization (127). Other studies, however, propose that MMF has no effect on Tregs or may facilitate the induction of a more tolerogenic environment (123).

Corticosteroids, such as dexamethasone and prednisolone, have been used for decades as basis for the treatment of inflammatory diseases and in patients post-organ transplantation. They regulate a wide spectrum of physiological processes and control not only inflammation but also carbohydrate and protein metabolism, fetal development, and behavior. For these reasons, despite their therapeutic efficacy, there are major drawbacks associated with the persistent use of glucocorticoids, such as osteoporosis and diabetes (128). However, in respect to the impact of these treatments on Treg therapy, many authors described positive effects of steroids on the maturation and expansion of Tregs. Glucocorticoids have been suggested to amplify the IL-2-dependent expansion of FOXP3^+^CD4^+^CD25^+^ T cells *in vivo* (129), increase FOXP3 expression by Tregs in patients affected by asthma (130), and restore the impaired suppressive function of Tregs in patients with relapsing multiple sclerosis (131). Furthermore, steroids may affect the inflammatory environment negatively controlling both Th1- and Th17-polarization in mice and humans (132–134).

This highlights the importance of strategies to tailor immunosuppressive therapy to ensure the *in vivo* survival of the injected Tregs or enhance their longevity *in vivo*. In this regard, the clinical protocol for ThRIL is based on a Treg-supportive immunosuppressive regimen including the use anti-thymocyte globulin (ATG), to induce lymphopenia with a preferential preservation of Tregs (135). Additionally, to limit memory T cell expansion post-ATG induction, patients are started on tacrolimus and prednisolone. One month prior to Treg infusion, in parallel with low-dose tacrolimus, the patients are given rapamycin, to promote selective Treg expansion *in vivo* (136). The intention behind this protocol: to create a tolerogenic milieu thus maximizing the potential efficacy of the exogenously administered Tregs through prolongation of their *in vivo* survival. It is also reassuring that these cells will be injected in a “Treg nurturing” environment, centered on the inclusion of rapamycin.

Thus, tailoring the immunosuppressive regimen along with the administration of *ex vivo*-expanded Tregs may potentially maintain post-liver transplant tolerance, accomplishing the ultimate aim of Treg immunotherapy trials in this setting.

## Stability and Longevity of the Injected Cells and Visualization *In Vivo*

For Treg cellular therapy to be a viable therapeutic avenue, two key factors need to be addressed. The first being that, following injection, the Tregs are stable in the graft and draining lymph nodes, irrespective of the local inflammatory environment following transplantation, and second, whether these cells are either long-lived or able to impart their tolerance to the host immune system.

As the function of Tregs is highly dependent on the constitutively high expression of FOXP3 (137), many groups have sought to find ways to stabilize its expression. As discussed earlier, epigenetic modification of the *FOXP3* locus has a major role in controlling *FOXP3* transcription, with demethylation of key regions correlated with suppressive function and lineage stability (138). In this regard, *in vitro* treatment with demethylating agents, such as azacytidine, have shown to promote the stability of FOXP3 expression in Tregs, resulting in the potent ability of these treated cells to protect from GvHD (139). In addition, a recent Phase I trial has shown that patients with acute myeloid leukemia, treated with azacytidine immediately after allogeneic stem cell transplantation, had a higher proportion of Tregs as compared to time-matched controls (140).

FOXP3 levels are not only regulated through transcriptional control but also through post-translational modifications. In the context of transplantation, most work has focused on acetylation of lysine residues, which is known to stabilize the FOXP3 protein (141, 142). It has been shown that inhibiting deacetylation with histone deacetylase (HDAC) inhibitors or genetically removing Sirtuin-1, a histone and protein deacetylase, leads to an improvement in Treg function and stability, ultimately leading to improved allograft survival (143). Thus, future directions of adoptive Treg cell therapy will necessitate further understanding of factors that cause Tregs to lose FOXP3 expression and ways to stabilize its expression.

The question of how long transferred Tregs survive *in vivo* is also of critical importance. It is understood that in order to establish long-term dominant tolerance, adoptively transferred Tregs must either survive and expand in the recipient, or be able to induce a tolerogenic phenotype on other T cells, a process known as infectious tolerance (144). It has been shown that some subpopulation of Tregs, such as those producing soluble factors, such as TGF-β (145), IL-10, and IL-35 (146), and the ongoing presence of recipient “infected” Tregs, are required to prevent allograft rejection (147, 148).

In the recent clinical trial of Treg therapy in hematopoietic stem cell transplantation, the transferred cells were no longer detected in the circulation after 2 weeks (56). Moreover, in the pediatric trial of Treg therapy in Type-I diabetes, infusion of 30 × 10^6^/kg polyclonally expanded Tregs resulted in doubling of the percentage of circulating Tregs and a trend of increase at 2 weeks (118). In these trials, it is not known whether the cells migrated to tissues or died. In this regard, we have recently used single photon emission computed tomography to image adoptively transferred Tregs in mice and reported that 24 h after intravenous injection, the cells were primarily localized in the spleen (149).

Therefore, to maximize the efficacy of Treg therapy, efforts will need to focus on finding ways to support the *in vivo* survival, engraftment, and function of the infused Tregs. Since Tregs depend on exogenous IL-2 for survival, a suggested approach has been to use low-dose IL-2, which lacks the toxicity and immunostimulatory effects of the higher IL-2 doses used to treat cancer patients (150). This approach has recently shown to increase the number of Tregs in patients with chronic GvHD (151), supporting the notion that low-dose IL-2 may be an ideal adjuvant to adoptive Treg cell therapy, by promoting Treg expansion in an otherwise inflammatory setting.

The future will also see studies defining the trafficking patterns of infused Tregs *in vivo*. In this regard, in a recent clinical trial of Treg immunotherapy in Type-I diabetes conducted at UCSF, Tregs were labeled with deuterium and their relative homing and survival period was recorded *in vivo* (*Bluestone* et al. *unpublished data*). In parallel, micro-PET computed tomography fusion has been used clinically to track infused T cells in the body and has further been refined to focus on distinct T cell populations, in particular Tregs (152). While these technologies are relatively new, the information gleaned from their inclusion in clinical trial protocols of Treg cell therapy will be invaluable, allowing for virtual visualization of these cells *in vivo*.

The future of cell therapy is also moving in such a way through cellular engineering, introducing concepts of traceable markers, tunable TCRs, chemotactic receptors to synthetic ligands, and drug inducible suicidal enzymes (153). These designer features would not only allow for the monitoring of infused Tregs, while also controlling their activities and trafficking patterns, but also for elimination if and when they become pathogenic (154, 155). Nonetheless, further advances in gene therapy would be required for these approaches to move forward, with licensing issues posing their own challenges and hurdles.

## Dose of Regulatory T Cells, Number of Injections, and Monitoring Outcomes

As in the ThRIL trial, the first trials of Treg therapy in solid-organ transplantation have started with a dose escalation study to assess the safety and tolerability of Tregs at various doses. It is anticipated that high Treg doses are needed for tolerance induction in view of pre-clinical studies in mouse models of transplantation where a high ratio of Tregs to Teffectors, in the order of 1:1–1:2, i.e., 33–50% of Tregs, is needed to prevent transplant rejection (29, 156). Moreover, it has been suggested that, combined with ATG induction, a single infusion of 3–5 × 10^9^ Tregs can effectively increase Treg percentage to more than 33% (157). One caveat is the use of antigen-specific Tregs, where studies have shown that lower numbers are needed to achieve the same functional efficacy as larger numbers of polyclonal Tregs (158, 159). Irrespectively, producing such large numbers of Tregs remains technically challenging, especially in view of studies showing a loss of FOXP3 expression after several rounds of stimulation. In this regard, more research is needed to understand Treg commitment and epigenetic regulation of FOXP3 expression so that the mechanisms can be harnessed to stabilize the Tregs.

Another point of consideration is if a single injection of Tregs is sufficient or whether multiple injections are required. This may be determined in larger Phase II efficacy studies, where patient outcomes should also be measured and an in-depth patient monitoring system planned. In this regard, molecular diagnostic tools can be utilized to assess a broad panel of biomarkers, associated with operational tolerance, to serve as surrogate end-points of efficacy (160–162).

In this regard, high-throughput, highly sensitive flow cytometric analysis can also be used to determine if the number of Tregs in the peripheral blood of recipients have increased or relatively quantify the composition of the T cell compartment following the intervention (163). Furthermore, the cytokine profile secretion capacity of these cells can be analyzed and thus their plasticity evaluated. Investigations using the complementarity-determining region 3 (CDR3) length distribution analysis can be used to explore the diversity of the TCR, in view of studies suggesting that the TCR repertoire might be a good predictor of graft outcome. In this regard, it has been suggested that the majority of kidney transplant patients with chronic rejection have an accumulation of oligo or monoclonal Vβ expansions while operationally tolerant recipients have a TCR repertoire like that of healthy individuals (164).

As such, a comprehensive immune monitoring plan of patients should be an integral part of a Treg therapy trial in order to gain mechanistic insight on the Treg function in patients. In addition, success in defining optimal ways of measuring tolerance would set the scene for subsequent trials in which accelerated drug minimization is the principal aim.

## Anticipated Cost and the Future

At present, the cost to manufacture a single “personalized” injection of Tregs in the CRF is over £20,000 in the UK. The data soon emerging on the safety of these cells in the setting of transplantation will provide the basis for progression to a larger Phase II/III study. The future progression of the cell therapy program will also see efforts focused on the optimization of the process development and potential commercialization of the cell-based therapies, through collaborations with industry and other organizations. It is anticipated that the future optimization of the manufacturing process for larger scale trials and commercialization would reduce the costs, making this modality of treatment broadly available and applicable in other disease settings.

## Conclusion

We are now entering an exciting era in the study of immunological tolerance. Several cellular and molecular strategies of tolerance induction have been developed in non-human transplant models that have shown considerable promise and are just now appearing in clinical trials. As such, the recent progress in Treg biology and the successes in the clinical grade manufacture of these cells has seen the start of clinical trials of Treg therapy in solid-organ transplantation. Such trials will provide the basis for progression to a larger Phase II/III study with a comprehensive patient immune monitoring plan and the use of biomarkers that can predict the successful induction of immune tolerance, allowing for the safe minimization/withdrawal of immunosuppression. With this all said, it is no secret that the panacea of immunological tolerance in transplantation is now ordained as we take steps ever closer to its fulfilment.

## Conflict of Interest Statement

The authors declare that the research was conducted in the absence of any commercial or financial relationships that could be construed as a potential conflict of interest.
